# Improved Cognitive Vigilance Assessment after Artifact Reduction with Wavelet Independent Component Analysis

**DOI:** 10.3390/s22083051

**Published:** 2022-04-15

**Authors:** Nadia Abu Farha, Fares Al-Shargie, Usman Tariq, Hasan Al-Nashash

**Affiliations:** 1 Biomedical Engineering Graduate Program, College of Engineering, American University of Sharjah, Sharjah P.O. Box 26666, United Arab Emirates; nadiaabufarha@gmail.com (N.A.F.); fyahya@aus.edu (F.A.-S.); utariq@aus.edu (U.T.); 2 Department of Electrical Engineering, College of Engineering, American University of Sharjah, Sharjah P.O. Box 26666, United Arab Emirates

**Keywords:** vigilance assessment, noise, feature extraction, dimensionality reduction, thresholds, wavelet transform, independent component analysis

## Abstract

Vigilance level assessment is of prime importance to avoid life-threatening human error. Critical working environments such as air traffic control, driving, or military surveillance require the operator to be alert the whole time. The electroencephalogram (EEG) is a very common modality that can be used in assessing vigilance. Unfortunately, EEG signals are prone to artifacts due to eye movement, muscle contraction, and electrical noise. Mitigating these artifacts is important for an accurate vigilance level assessment. Independent Component Analysis (ICA) is an effective method and has been extensively used in the suppression of EEG artifacts. However, in vigilance assessment applications, it was found to suffer from leakage of the cerebral activity into artifacts. In this work, we show that the wavelet ICA (wICA) method provides an alternative for artifact reduction, leading to improved vigilance level assessment results. We conducted an experiment in nine human subjects to induce two vigilance states, alert and vigilance decrement, while performing a Stroop Color–Word Test for approximately 45 min. We then compared the performance of the ICA and wICA preprocessing methods using five classifiers. Our classification results showed that in terms of features extraction, the wICA method outperformed the existing ICA method. In the delta, theta, and alpha bands, we obtained a mean classification accuracy of 84.66% using the ICA method, whereas the mean accuracy using the wICA methodwas 96.9%. However, no significant improvement was observed in the beta band. In addition, we compared the topographical map to show the changes in power spectral density across the brain regions for the two vigilance states. The proposed method showed that the frontal and central regions were most sensitive to vigilance decrement. However, in this application, the proposed wICA shows a marginal improvement compared to the Fast-ICA.

## 1. Introduction

Vigilance level assessment is of paramount importance to prevent human failure in different work environments [1]. Vigilance assessment in real-time is very important to maintain high cognitive efficacy and avoid human error [2,3]. In real-life applications, it was observed that workload, stress, time-on-task, and drowsiness are major factors that contribute to vigilance decrement [3,4,5]. Researchers have reported that maintaining vigilance in a stressful environment requires hard mental work [2]. The performance of individuals in given tasks significantly decreased with time [6]. According to a recent survey [7], 74% of European drivers experience fatigue when they are behind the wheel, which could lead to accidents. The case was similar for drivers in North America (69%), Africa (64%), and Asia-Oceania (53%).

There are several methods to assess vigilance: subjective, behavioral, and objective assessment methods [8]. Assessing vigilance using the objective method is better than the other two methods as it helps in evaluating the operator’s mental state while performing a task as a function of time. Many studies have proposed and designed new objective methods for vigilance assessment. This includes methods such as heart rate variability [9], galvanic skin response [10], pupil diameter, eye blink frequency [8], and neuroimaging modalities [11]. All these modalities are considered useful in cognitive workload assessment, yet some limitations may reduce the assessment reliability.

One of the modalities used for vigilance assessment is the electroencephalogram (EEG). The EEG possesses several advantages as it is non-invasive, less expensive, and safe for long-term monitoring [12,13]. A study in [14] compared the detection accuracy using subjective ratings and EEG-based methods. The study examined the workload using EEG signals at various levels of workload. The entropy, power, and wavelet coefficients were obtained from the signal. The EEG signal performance outperformed the self-ratings with an accuracy of 98% for the differentiation between seven load levels, in contrast to 31% for the self-rating. Another study [15] has utilized the EEG to detect fatigue for high-speed train safety by tracking the driver’s vigilance level with a wireless EEG. The study achieved a classification accuracy of 90.70% for driver vigilance detection using a support vector machine classifier.

Although the EEG has a high temporal resolution, it is usually contaminated by undesired artifacts [16]. Artifacts are either well-localized in frequency, which impacts the whole-time signal, or may appear in a narrow temporal segment with an effect on the original frequency spectrum [17]. In particular, EEG artifacts may be due to the measurement devices or to human physiology [18]. The artifacts raised from the measurement devices are the results of power line interference, faulty electrodes, or high electrode impedances. These types of artifacts can be avoided by utilizing good practices in circuit design and experimental procedures. Meanwhile, physiological artifacts such as eye movements, eye blinks, and cardiac and muscle activity remain a challenge to be removed. The presence of artifacts makes the analysis of the EEG difficult for clinical evaluation [18]. Hence, extensive preprocessing is a very important step for mining EEG data [19]. EEG preprocessing mostly focuses on bad channel/epoch identification and removal. In addition, referencing and applying high-pass filtering is a key step [20].

Classic filtering of artifacts can enhance the quality of EEG signals, but artifact removal still requires some advanced signal-processing methods. Independent Component Analysis (ICA) is a very effective method for removing physiological interference. It is well established and helps in removing artifacts and enhancing the primary brain components [21]. A variety of pipeline-type toolboxes that are used for artifact detection and removal are available, such as the EEGLAB toolbox [22]. For example, ADJUST (automatic EEG artifact detector based on the joint use of spatial and temporal features) [23], utilizes ICA to cancel features that are associated with various stereotypical artifacts, such as eye blinks, eye movements, and discontinuities. On the other hand, the FASTER (fully automated statistical thresholding for EEG artifact rejection) pipeline [24] employs compound statistical thresholding and ICA to remove bad channels/epochs. Corrupted channels are usually associated with eye movements, muscle artifacts, and white noise. A study in [25] showed that the signal-to-noise ratio could be improved by applying the FASTER pipeline and ordinary average referencing. The study also introduced a multistage robust referencing scheme that deals with noisy channel-reference interaction. The preprocessing pipeline used in [25] showed more uniform statistical behavior for various headsets and experimental paradigms. Another study in [26] utilized a combination of ICA and discrete wavelet transform (DWT) for artifacts cancellation on Electromyography (EMG) data to overcome the limitations of individual methods. The proposed approach for the joint use of both ICA and DWT showed very good ability in separating the original signal from the artifacts, especially in the case of corrupted signals. In addition, a study in [27] investigated the practicality of using a wavelet transform in preprocessing the EEG data. The study achieved a high classification accuracy of 91.4% in detecting spikes using the Daub-20 wavelet function. Recent vigilance and emotion studies [28,29] used ICA with visual inspection to eliminate noise, eye blinks, and movement artifacts. The studies identified and removed 2-frontal ICs that were highly associated with artifacts and reconstructed the rest of the ICs to form the cleaned EEG signals. Another study in [30] developed the Automagic standardized preprocessing pipeline to assess all currently available preprocessing methods and examine the effect of applying combinations of preprocessing approaches on EEG data. The Automagic method showed that using algorithms to detect channels associated with artifacts in combination with a multiple artifact rejection algorithm, which is ICA-based, is very effective in artifacts rejection.

This work aims to investigate the accuracy of vigilance level assessment obtained using different preprocessing methods such as ICA and wICA. The preprocessed EEG signals are then used to classify the cognitive vigilance levels. The power spectral density was the main feature extracted from the EEG recording and utilized for the vigilance level assessment by employing five different classifiers: K-Nearest Neighbors (KNN), Discriminant Analysis (DA), Naive Bayes (NB), Decision Tree (DT), and Support Vector Machine (SVM). This paper is structured as follows: Section 2 covers the methodology and Section 3 presents the features extracted from the EEG signal. Next, the results and the discussion are covered in Section 4 and Section 5, respectively. Finally, the conclusions are added in Section 6.

## 2. Methodology

### 2.1. Participants

In this study, nine healthy volunteer students from the American University of Sharjah, aged 24.5 ± 5.5 years, were enrolled in the experiment. All participants met the predefined inclusion criteria where they have normal hearing, normal or corrected-to-normal vision, no history of psychiatric or cognitive disorders, no symptoms of drug addiction or abuse, and no intake of long-term medications. Participants were informed of the experiment procedure and nature. Each participant gave their informed written consent. The experiment was performed between 3:00 p.m. and 7:00 p.m. to reduce the effects of circadian rhythm on vigilance levels. The experiment protocol was organized based on the declaration of Helsinki and was accepted by the Institutional Review Board of the American University of Sharjah (Protocol Code 19-513, date of approval 31 March 2020).

### 2.2. Vigilance Task

The nine subjects performed a 45 min computerized Stroop Color–Word Task (SCWT). The SCWT was set to display six basic colors: blue, green, red, magenta, cyan, and yellow. A word with a specific color was displayed each time and the answers that match the color of the word were displayed in a random sequence. The displayed color word does not represent its true meaning, in such a way that the correct answer is the color of the word but not its meaning. For example, if blue is written in green, then green is the correct answer [31]. The results were acquired from the participants by clicking the left mouse button on one of the six color options that appeared on the screen. The experimental difficulty was increased using random colors for the background of the answering options. During the training stage, the reaction was recorded to determine the maximum time for each trial. The participants received a feedback message of “correct “or “incorrect” for every trial in addition to the recorded reaction time. Participants also received a feedback message of “Time is up” when they consumed the time given for the trial without a response.

**Figure 1:**
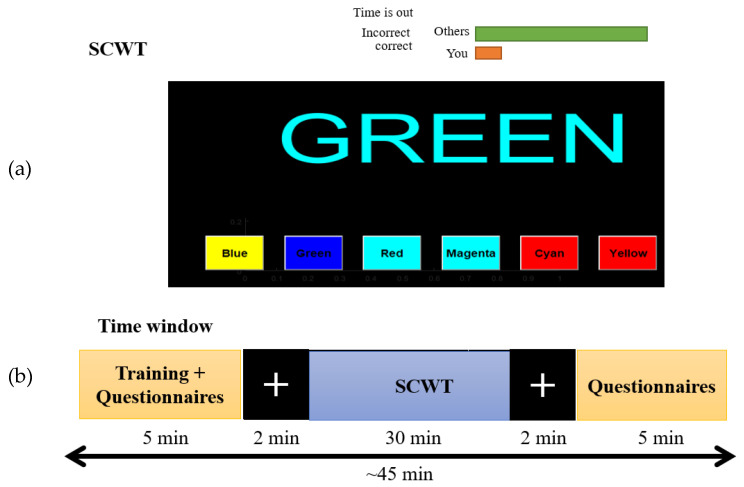
Experimental protocol (**a**) Stroop Color–Word Task (SCWT) presentation interface, and (**b**) timing window. In the timing window, the plus sign on the black background is for the pre- and post-baseline.

The EEG data recording followed several steps. First, the participants were asked to fill out a Brunel Mood Scale questionnaire before starting the recording [32]. Second, participants were asked to perform the task for three minutes to familiarize themselves with the nature of the SCWT. Third, participants wore the EEG cap and performed the SCWT for 30 min. In addition, subjects were asked to reduce their head movements in order to obtain good-quality EEG data. Finally, all participants performed the same questionnaires again. Figure 1 shows the experimental protocol of the SCWT with a time window of 45 min [31].

### 2.3. Experimental Setup and Data Acquisition

**Figure 2:**
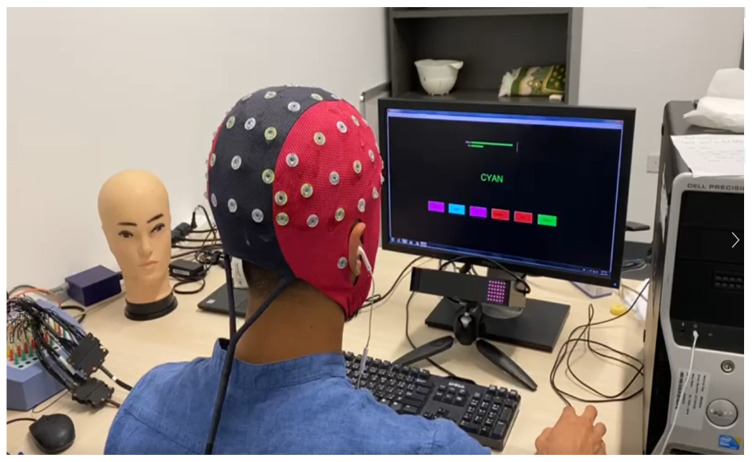
EEG data acquisition and experimental setup.

The experiment was performed in the Biomedical Engineering Laboratory at the American University of Sharjah. The lab is a quiet place, where light and temperature can be controlled. The EEG data were obtained using 64-Ag/AgCl scalp EEG electrodes (ANT Neuro EEG system), at a sampling rate of 500 Hz. The impedance of each electrode was reduced and maintained below 10 KΩ by applying an amount of conductive gel layer between the electrode and the scalp. The AFz electrode was set as the system ground, and the mastoid electrodes M1 and M2 were used to reference the rest of the electrodes, as shown in Figure 2.

### 2.4. EEG Data Preprocessing

Wavelet Analysis is a frequency representation that provides better temporal resolution for the components with a high frequency, and better frequency resolution for the lower-frequency components [33]. The Discrete Wavelet Transform (DWT) is very important in providing the input matrix for the ICA approach. DWT works by decomposing the signal into two phases known as the detail phase and approximation phase [34]. Applying ICA directly would provide spectral improvement because of suppressing the typical artifacts, but this suppression may lead to corrupting the spectrum of the underlying neural activity. The proposed method, combining WT and ICA which we call wICA, allows us to perform artifact removal when the recordings are non-redundant. In addition, it allows us to perform artifact removal on a wider range of corrupted recordings. The Daubechies DWT algorithm was applied to each channel of the multichannel recordings separately. This algorithm has the ability to distinguish the finer details in the frequency domain signal [35], in addition to achieving high classification accuracy in many applications [36]. Prior to wICA, the EEG signal was notch-filtered at 50 Hz to remove the power line interference [29,37,38], and bandpass-filtered between 0.1 and 40 Hz [39,40]. The wICA method includes the following steps:

1. In standard ICA analysis [26,31], the recorded EEG signal matrix, *X*(*t*)*,* is modeled by the source components *s*^T^ = [$s_{1}$, $s_{2}$, …, $s_{n}$], where *n* is the source number, which are assumed to be statistically independent, mixed using the mixing matrix *M* to produce the EEG signals:
  (1) X(t)=Ms(t)
  After identifying the artifact components, they can be set to equal zero. Artifact removal will be followed by ICA signal reconstruction. The reconstructed EEG is:
  (2) $$\hat{X}\left(t\right)=M\hat{s}\left(t\right)$$
2. In wICA, the artifact components are identified and subtracted from the signal to keep the neural components, unlike the conventional ICA artifact removal which sets all artifact components to zero. Let us assume an independent component $s_{1}\left(t\right)$ which is a combination of a high amplitude artifact f(t) and a low amplitude neural signal l(t) such that:
  (3) $$s_{1}\left(t\right)=f\left(t\right)+l\left(t\right)$$
3. The features of the signals f(t) and l(t) can be estimated. The artifact f(t) has high power that is localized in both time and frequency domains, whereas l(t) has low power. Then, by estimating f(t), we can subtract it to reconstruct clean data as follows:
  (4) $${\hat{x}}_{j}\left(t\right)=a_{j1}\left(l\left(t\right)\right)$$
  where $a_{j1}$ is the corresponding weight from the mixing matrix *M*, and the artifact-free signal ${\hat{x}}_{j}\left(t\right)$ is the ICA-corrected EEG signal.
4. To separate the artifact from the neural information in *s*_1_, we apply wavelet transformation on the independent components to obtain the wavelet coefficients ${\left\{W\left(j,k\right)\right\}}_{s_{i}}$, where j and k are integers that play a role in the decomposition level and the temporal localization. The wavelet coefficients are then thresholded by setting the coefficients more than a certain threshold to zero. This way, the coefficients representing the artifact are removed. Thresholding was optimized following the techniques found in [39]. The threshold value, T is defined by T =$\;\sqrt{2\log N\sigma }$, where *N* is the length of the data segment to be processed, and σ^2^ is the estimator of the variance of the neural wideband signal part.
5. The remaining wavelet coefficients are then used to reconstruct the independent components *s*_1_, which are predominantly neural signals. Steps 3 to 6 are repeated for all independent components.
6. We then construct the wICA-cleaned EEG using the demixing matrix and the independent components:
  (5) $$\hat{X}\left(t\right)=M\left[l_{1},\;l_{2},\;\ldots ,\;l_{n}\right]\left(t\right)$$

## 3. Feature Extraction

Vigilance level reflects the interaction between brain regions that helps us in understating the cognitive process in the brain. First, we defined two vigilance states for the subjects within the 30 min EEG recordings: the alert state, including the first 5 min of EEG signals, and the vigilance decrement state, which refers to the last 5 min of EEG signals within the SCWT. The criteria for this decision were based on behavioral data analysis as reported in our previous study [28]. Second, the clean EEG signals were analyzed using the fast Fourier transform method to extract the Power Spectral Density (PSD). We extracted the PSD across different EEG frequency bands, namely the delta (0.1–4 Hz), theta (4–8 Hz), alpha (8–13 Hz), and beta (13–30 Hz) [40] bands. In this context, the PSD was calculated using a moving window of two seconds within each of the five-minute windows. The total PSD features extracted from the two vigilance states per subject correspond to 62 electrodes with 300 data points each. These features were then used as input to the classifiers.

Various classification methods are used in machine learning, including Naive Bayes (NB), Random Forest, K-Nearest Neighbors (KNN), and Decision Tree (DT). Support vector machine (SVM) classifiers are also considered effective in binary classification. We have employed five classifiers, namely KNN, Discriminant Analysis, Naive Bayes, Decision Tree, and SVM to distinguish between the two vigilance levels. The selected classifiers are widely recognized in the field of brain–computer interfaces for being fast and reliable [28]. KNN is known for training in a fast manner, SVM provides high accuracy, and Naive Bayes is fast and can be used to make real-time predictions [41]. We are employing five different classifiers to compare their performance to classify vigilance levels. Our proposed approach can also help researchers in selecting classifiers when dealing with EEG data for vigilance assessment.

**Figure 3:**
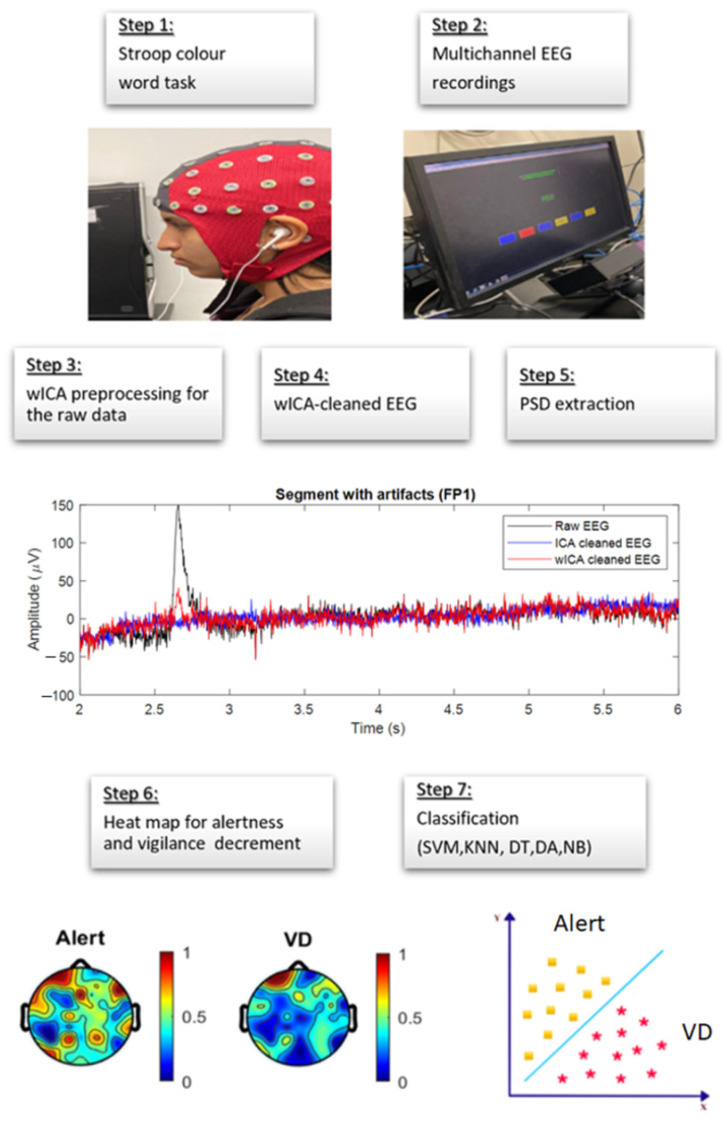
Flow chart for EEG data analysis using wICA in vigilance level assessment. The yellow squares and asterisks represent alertness and vigilance decrement in the feature space.

Figure 3 illustrates the framework of data processing from the data collection, preprocessing, feature extraction, and vigilance level assessment using the five different classifiers, showing the steps of the preprocessing applied separately to two data sets. One dataset was ICA-cleaned, and the other one was wICA-cleaned.

## 4. Results

### 4.1. Artifact Removal and PSD

**Figure 4:**
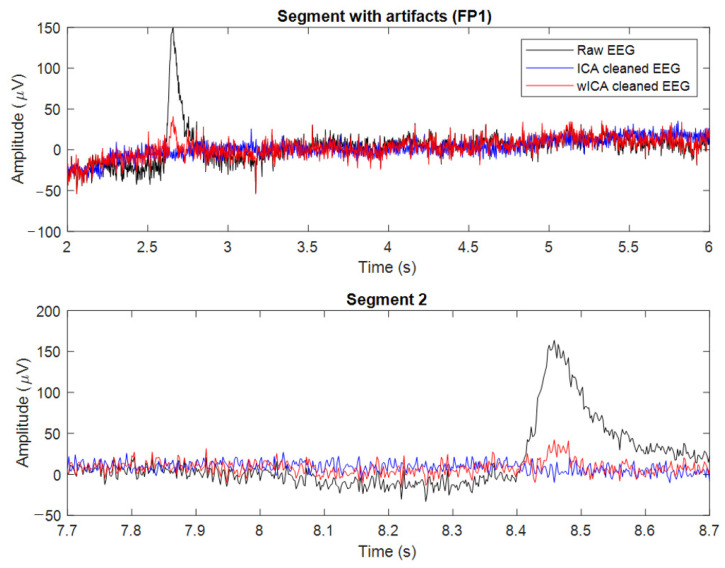
wICA-cleaned EEG from subject 8 for time segments of 4.0 and 1.0 s, respectively.

The EEG was recorded during the experiment for 30 min; two windows of the recording were extracted, with a length of 5 min each. The first five minutes of the recording represent the alertness state, and the last five minutes represent the vigilance decrement state. The data preprocessing was applied only on the windows of interest. To illustrate the preprocessing of the EEG data using wICA, we have plotted the raw EEG data, the ICA-cleaned EEG data (using the Fast-ICA algorithm [39]), and the wICA-cleaned EEG data for two different time segments, in Figure 4. The first and second time segments are for 4 s and 1 s recordings, respectively. FastICA leads to underestimated neural activity, whereas the wICA technique preserves the power and the characteristics after artifact suppression.

### 4.2. PSD

**Figure 5:**
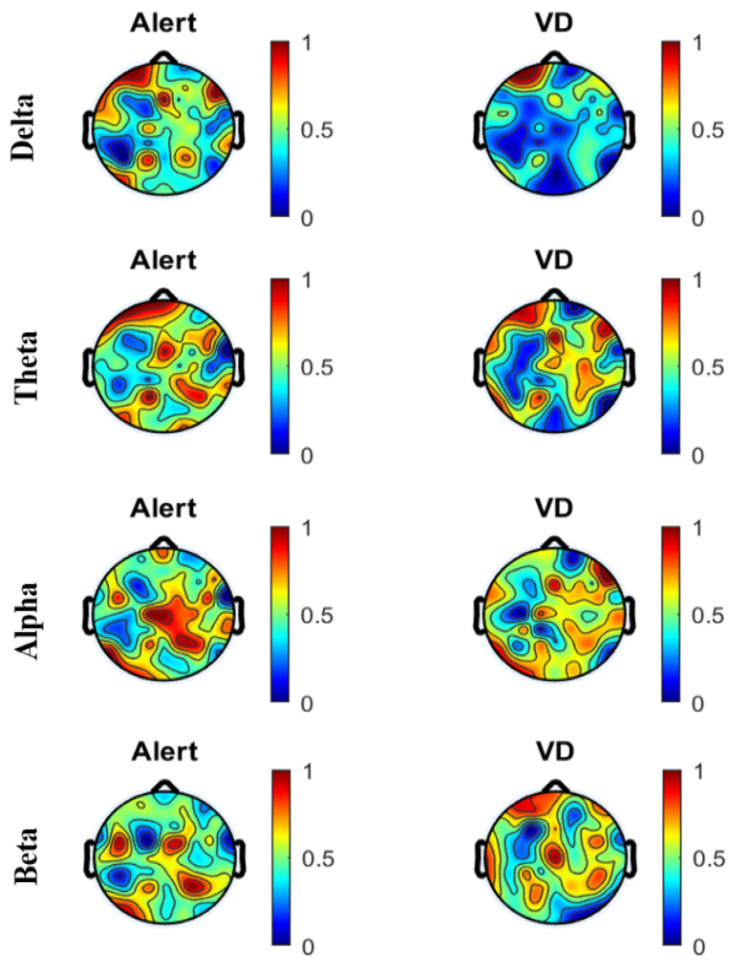
Comparison of PSD for all subjects under the two mental states, alert and vigilance decrement, in the four frequency bands using ICA-cleaned EEG.

**Figure 6:**
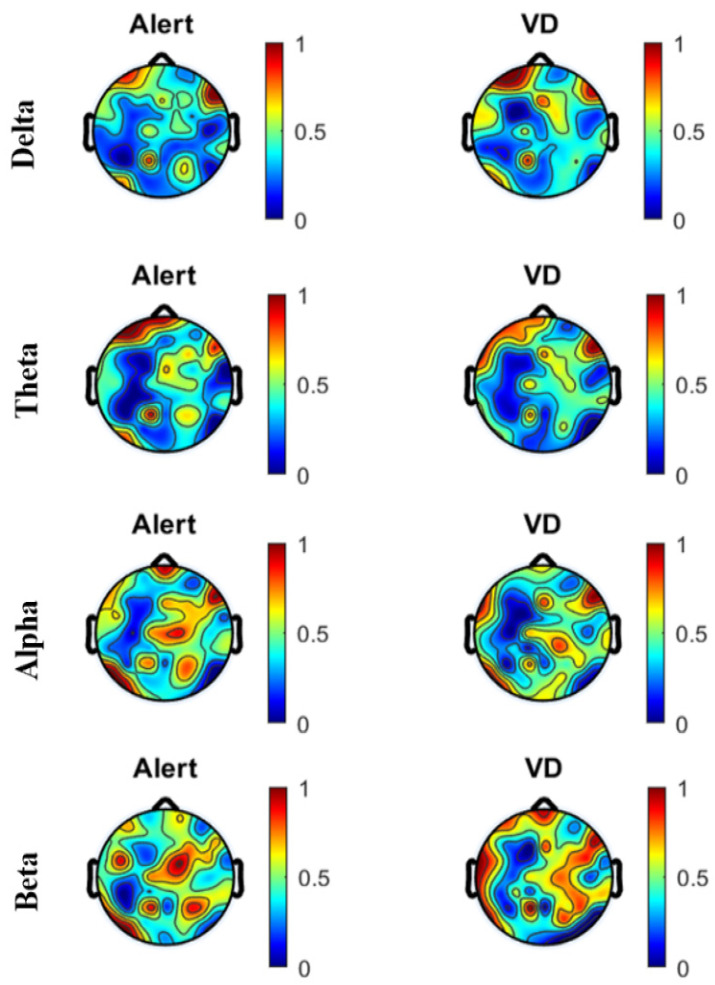
Comparison of PSD for all subjects under the two mental states, alert and vigilance decrement, in the four frequency bands using wICA-cleaned EEG.

A comparison of the Power Spectral Density (PSD) using a topographical map was made for the subjects under the two mental states, alert and vigilance decrement, for each of the four frequency bands. Figure 5 shows the topographical map created using the ICA-cleaned EEG data, and Figure 6 shows the topographical map created using the wICA-cleaned EEG data.

The SWCT requires high attention in recognizing colors in addition to a strong memory for faster responses due to less reaction time. Figure 5 and Figure 6 highlight the change in the PSD across the areas of the brain between the alertness states and vigilance decrement, for both the ICA- and wICA-cleaned EEG. The occipital and central brain regions were most sensitive to vigilance decrement. Note that the occipital brain region is linked to the processing of visual activity, memory formation, distance, and depth perception, in addition to being responsible for color determination [42]. The central brain region is responsible for information processing [42]. By comparing Figure 5 and Figure 6, we can see that the wICA-cleaned EEG showed higher sensitivity to the task. We can also see that with increasing time spent on the task, more regions appeared to be sensitive to vigilance decrement.

### 4.3. Classification

Data from both 4 and 1 s windows were extracted from the ICA- and wICA-cleaned EEG for alertness and vigilance decrement. The EEG data were classified in a subject-dependent fashion, where a randomized 10-fold cross-validation approach was performed using features extracted both from the wICA-cleaned and Fast-ICA-cleaned EEG data. In the 10-fold cross-validation, each of the EEG feature sets was divided into ten subsets. Nine of these subsets were used for classifier training, whereas the last subset was used for the estimation of classification accuracy, sensitivity, and specificity. This process was performed ten times such that all subsets had an opportunity to be used as testing data. We tested five different classifiers (KNN, Discriminant Analysis, Naive Bayes, Decision Trees, and SVM). Table 1 and Table 2 compare the classification accuracy, specificity, and sensitivity of the Fast-ICA- and wICA-cleaned EEG for the two cognitive states. Table 1 shows the results for the Fast-ICA-cleaned EEG data. Here, all bands showed similar classification accuracy for the vigilance assessment when the Discriminant Analysis classifier was utilized. When other classifiers are used, the Beta band outperforms all other EEG frequency bands. Table 2 shows the results for the wICA-cleaned EEG data. It shows higher classification accuracy across all bands when compared with Table 1. Similar to Table 1, Table 2 also reports the classifier performance with respect to the frequency bands. It is worth noting that Discriminant Analysis outperformed the other classifiers for both the ICA-cleaned EEG and the wICA-cleaned EEG vigilance level classification. Discriminant Analysis also showed a slightly better classification accuracy in Table 1 compared to Table 2.

Table 3 compares the vigilance assessment accuracy based on the power spectral density between our study and [28]. The comparison is between the three EEG preprocessing approaches used for the vigilance assessment: wICA and Fast-ICA were adopted in this study, and traditional ICA was used in [28]. The accuracies below are the results of the SVM classification between alertness and vigilance decrement.

## 5. Discussion

In this paper, we have investigated vigilance level assessment using two differently preprocessed EEG data, where the preprocessing approach combines both the independent component analysis and the wavelet transform. Our study used both the ICA and the wICA techniques and investigated the performance of five different classifiers for classifying vigilance levels. Our study has demonstrated that the wICA-cleaned EEG showed higher accuracy for vigilance assessment compared with the ICA, as seen in Table 1 and Table 2. The accuracy was higher across all classifiers except the Discriminant Analysis, which showed slightly higher accuracy, as seen in Table 1. The reason may be that the algorithm we have utilized works by subtracting the artifacts instead of performing ICA artifact suppression. Discriminant Analysis provided the highest accuracy among all classifiers, followed by the SVM classifier. It is worth mentioning that [28] has also compared different classifiers for vigilance assessment based on power spectral density, and SVM surpassed the other classifiers for a subject-dependent classification. Many studies have utilized EEG for vigilance assessment. Refs. [44,45,46] showed that performance decrement was associated with a decrease in the level of vigilance. These studies have reported an increase in theta- and alpha-band activity and a decrease in beta-band activity for the EEG power spectral density. On the other hand, Refs. [47,48,49] discussed the brain regions that are more sensitive to the transition period from wake to sleep; these studies have reported that major changes were detected in the posterior regions of the brain (cortical, parietal, and occipital scalp localization). Studies [50,51,52] reported that the alpha band plays a significant role during vigilance decrement, which was mostly observed in the occipital cortex. Our results using wICA demonstrated that the occipital and central brain regions were most sensitive to vigilance decrement, which complies with what has been reported in previous studies.

Different preprocessing techniques have been utilized for cognitive workload assessment using EEG data. A study in [15] has used a wavelet de-noising method for the EEG data preprocessing with the target of vigilance assessment based on the power scalp topographies classified using the SVM classifier, and reported a classification accuracy of 90.70% for fatigue detection. The authors of [53] employed a conservative Hampel outlier filter to reduce the occasional impulse-like artifact in the EEG signal. This study presented a user-state detection system in an active virtual reality environment based on a fourfold classification. The EEG spectral amplitudes for the alpha and beta frequency bands showed classification accuracy reached 81.1% for different task levels. In [54], the authors adopted the Principal Component Analysis (PCA) algorithm for artifact removal and reduced the signal dimensionality for the purpose of vigilance assessment. The assessment of vigilance was based on the power spectral density and was classified by an SVM classifier. The PCA appeared to help in achieving higher accuracy in the alpha and beta bands, but only a few of the nine subjects showed a significantly high classification accuracy. Study [28] targeted vigilance assessment based on the power spectral density and classified by the SVM classifier. The authors used traditional ICA for cleaning the EEG data, whereas we have employed both Fast-ICA and wICA cleaning. Using an SVM classifier, we were able to outperform this study, showing higher classification accuracy for all frequency bands, as presented in Table 3. Even though the accuracy did not increase significantly between the ICA and wICA techniques, the wICA algorithm is a fully automatic wavelet-based component correction method, in addition to being an important step towards the development of accurate, reliable, and automatic EEG artifact-removal methods. Utilizing wICA in different cognitive workload assessments could help in enhancing classification accuracy.

For various applications, it is necessary to detect and mitigate EEG artifacts. These artifacts, if not removed, could cause a significant discrepancy in the results, e.g., in brain source localization [55]. Developing an algorithm to mitigate these artifacts properly is very important. Therefore, preprocessing algorithms that eliminate the artifacts without removing any activity of interest are considered effective preprocessing approaches. Many efforts in EEG preprocessing have been made by employing the ICA signal decomposition. Despite the fact that ICA is considered an important preprocessing approach, its results can be enhanced [56]. It is worth noting that ICA-cleaned EEG may indeed reduce the spectral presence of typical artifacts [57], but they may also cause the distortion of cerebral activities in EEG recording [58]. wICA enhances ICA preprocessing by improving the quality of artifact suppression and enhancing the performance of ICA.

The wICA approach helps in improving the common ICA artifact suppression method. It has been demonstrated in many studies using independent component analysis that ICA is time-consuming as it may be dependent on manually choosing the ICs that are related to artifacts with a visual inspection. The ICA approach may also suffer from being subjected to human bias and it may not be considered effective for real-time applications [59]. Whether the ICs rejection occurs manually or automatically, ICA does not guarantee that no cerebral activity is leaked into the rejected ICs. If any cerebral activity is leaked into the rejected ICs, this approach will lead to the loss of some desired information [59]. A study [60] showed that ICA could be successfully employed in artifact removal only if the signals have a small degree of non-Gaussianity. The study has also demonstrated that when using ICA, if the number of recordings is less than the total number of signal sources (including artifact sources), ICA will only be able to separate the components with relatively high magnitude. ICA also has some unavoidable issues, for example, the order of the independent components will change with every estimation and cannot be determined beforehand [60]. The authors of [61] also discussed the variability associated with ICA uncertainty and how it influences the results of the clean EEG. The ICA approach is also dependent on reducing higher-order statistical dependencies. It was shown in [62] that, given the same data, ICA decompositions alter with each trial and return different solutions.

The joint use of a discrete wavelet transform besides ICA enhances the artifact suppression in the EEG signal. This approach uses the wavelet thresholding to conserve the time-frequency structure of the artifacts by denoising the demixed ICs. The results allow us to recover any cerebral activity that may have been leaked into the ICs related to artifacts. In [39], the authors compared EEG preprocessing using both ICA and wICA techniques. The study showed an increment in the power density amplitude for the wICA method compared with ICA alone. The same study showed that both ICA and wICA preprocessing techniques would suppress the ocular and heart artifacts, but the wICA method is superior in preserving the EEG cerebral parts compared with the ICA method. In [63], an improved Fast-ICA algorithm was enlisted. The improvement included adopting the wavelet packet energy spectrum for extracting the feature information in the separated samples. The results showed that the improved Fast-ICA and wavelet packet energy method outperformed the classic Fast-ICA in convergence speed and separation effect, in addition to being very effective in feature extraction. Studies [64,65] worked on extracting and classifying motor unit action potentials for electromyography signal decomposition. The approach utilized in the study included combining independent component analysis and a wavelet filtering method for removing the power interference component from EMG recordings. This technique appeared to be fast, robust, and took less time for the motor unit action potential extraction than the traditional method.

In line with this, our results demonstrate that the wICA method has outperformed the ICA method. In the delta, theta, and alpha bands, the mean accuracy in wICA was 96.9% whereas it was 84.66% using ICA. However, no significant improvement was observed in the beta band. In addition, we compared the topographical maps to show the changes in power spectral density across the brain regions for the two vigilance states. The wICA method showed that the frontal and central regions were most sensitive to vigilance decrement. However, in this application the proposed wICA has marginal improvement compared to the Fast-ICA, which could be due to shift sensitivity, poor directionality, and lack of phase information of the wavelet transform.

## 6. Conclusions

In this paper, we have shown that wavelet ICA can lead to improved classification accuracy when applied to vigilance level assessment. The methods we have presented in the paper are divided into two sections: first, applying an independent component analysis, and second, applying a discrete wavelet transform. The joint use, termed wICA, provided a better preprocessing output for the EEG signal. The wICA approach reduced the distortion in the signal and, in addition, recovered a substantial neural signal that was lost within the artifacts as shown in Figure 4. The wICA-cleaned EEG was used to assess vigilance based on the mean power spectral density of the four extracted bands. The delta, theta, and alpha bands showed high classification accuracy between the two mental states, alertness and vigilance decrement. The classification was carried out using five different classifiers, where Discriminant Analysis and SVM showed the highest classification accuracy. Topographical maps were created using the PSD for both the ICA-cleaned and the wICA-cleaned EEG. The maps showed that the frontal and central brain regions were most sensitive to vigilance decrement. In addition, from Figure 5 and Figure 6 we can see the color change in the cortical map, which indicates that wICA removed the unwanted noise.

## Figures and Tables

**Table 1:** The accuracy of subject-independent Fast-ICA-cleaned EEG-based vigilance classification.

	Band	Delta	Theta	Alpha	Beta
Classifier					
Accuracy					
SVM		95.4 ± 1.8	96.4 ± 1.7	96.0 ± 2.5	97.5 ± 1.8
KNN		91.6 ± 3.4	91.7 ± 3.4	89.3 ± 5.3	92 ± 5.6
DT		84.0 ± 5.7	85.5 ± 5.5	84.8 ± 4.6	88.7 ± 6.6
DA		99.3 ± 0.2	99.5 ± 0.4	99.5 ± 0.4	99.4 ± 0.3
NB		80.0 ± 5.7	82.3 ± 6.6	82.6 ± 7.6	87.3 ± 7.3
Specificity					
SVM		94.5 ± 2.4	95.5 ± 2.0	96.6 ± 2.4	97.0 ± 2.5
KNN		91.8 ± 4.1	93 ± 3.2	91.5 ± 5.6	92.1 ± 7.2
DT		85.0 ± 5.6	85.4 ± 4.7	83.6 ± 4.9	88.7 ± 6.4
DA		99.3 ± 0.5	99.4 ± 0.7	99.5 ± 0.5	99.5 ± 0.3
NB		79.3 ± 4.1	83.7 ± 5.1	80.3 ± 11	87.9 ± 5.7
Sensitivity					
SVM		96.3 ± 2.5	97.3 ± 2.1	95.5 ± 3.9	98.1 ± 1.4
KNN		91.4 ± 5.1	90.5 ± 5.6	87.2 ± 7.4	92.2 ± 7.5
DT		83.0 ± 5.6	85.5 ± 4.7	85.9 ± 4.8	88.7 ± 6.4
DA		99.3 ± 0.3	99.6 ± 0.4	99.6 ± 0.4	99.4 ± 0.9
NB		80.7 ± 9.5	81.8 ± 9.6	84.8 ± 7.7	86.7 ± 9.5

**Table 2:** The accuracy of subject-independent wICA-cleaned EEG-based vigilance classification.

	Band	Delta	Theta	Alpha	Beta
Classifier					
Accuracy					
SVM		96.1 ± 2.6	97.6 ± 0.8	97.0 ± 1.3	98.3 ± 0.8
KNN		92.2 ± 4.8	92.1 ± 4.5	90.5 ± 3.8	95.4 ± 4.0
DT		85.1 ± 7.4	86.4 ± 5.3	82.5 ± 6.2	89.2 ± 5.4
DA		99.3 ± 0.2	99.4 ± 0.1	99.4 ± 0.2	99.3 ± 0.5
NB		82 ± 6.8	85 ± 6.8	82.9 ± 8.5	89.3 ± 6.0
Specificity					
SVM		95.8 ± 3	97 ± 1.7	97.2 ± 1.5	98.2 ± 0.8
KNN		93.7 ± 5.5	93.9 ± 4.8	92.6 ± 4.2	96.2 ± 4.5
DT		85.4 ± 7.1	87.2 ± 6.1	82.4 ± 6.5	89.3 ± 4.9
DA		99.3 ± 0.2	99.5 ± 0.3	99.5 ± 0.3	99.6 ± 0.3
NB		82.6 ± 8.6	85.3 ± 8.0	82.6 ± 12.5	91.6 ± 4.6
Sensitivity					
SVM		96.4 ± 2.6	98.3 ± 0.6	96.9 ± 2.2	98.4 ± 1.4
KNN		90.7 ± 5.7	90.4 ± 5.7	88.4 ± 5.1	94.5 ± 5.5
DT		84.7 ± 8.1	85.7 ± 4.7	82.6 ± 6.6	89 ± 6.1
DA		99.2 ± 0.4	99.3 ± 0.2	99.3 ± 0.2	99 ± 1.0
NB		81.3 ± 8.1	84.7 ± 6.8	83.2 ± 9.6	87 ± 8.4

**Table 3:** The SVM classification accuracy for each EEG frequency band.

EEG Frequency Band	Delta	Theta	Alpha	Beta
wICA -SVM classification accuracy	96.1 ± 2.6	97.6 ± 0.8	97 ± 1.3	98.3 ± 0.8
Fast ICA-SVM classification accuracy	95.4 ± 1.8	96.4 ± 1.7	96.0 ± 2.5	97.5 ± 1.8
ICA-SVM classification accuracy [43]	87.9 ± 9.5	82.8 ± 12.8	83.3 ± 13.4	96.9 ± 2.2

## Data Availability

Raw EEG data can be requested through a formal email.
